# Syphilis

**Published:** 1846-05

**Authors:** 


*Syphilis.*—M. Cullerier has lately performed a series of experiments in order to determine the inoccubility of the lower animals, with the syphilitic poison. The result of repeated trials upon monkeys, Guinea-pigs, &c., appears to be that the disease in question is confined to the human race, as in no instance was the experimenter enabled to communicate the disease.—*Half-Yearly Abstract.*

